# Fuzzy Logical Algebras and Their Applications

**DOI:** 10.1155/2015/682648

**Published:** 2015-03-25

**Authors:** Jianming Zhan, Bijan Davvaz, Wieslaw A. Dudek, Young Bae Jun, Hee Sik Kim

**Affiliations:** ^1^Department of Mathematics, Hubei University for Nationalities, Enshi, Hubei 445000, China; ^2^Department of Mathematics, Yazd University, P.O. Box 89195-741, Yazd, Iran; ^3^Institute of Mathematics and Computer Science, Wroclaw University of Technology, 50-370 Wroclaw, Poland; ^4^Department of Mathematics Education, Gyeongsang National University, Jinju 660-701, Republic of Korea; ^5^Department of Mathematics, Hanyang University, Seoul 133-791, Republic of Korea

It is well known that an important task of the artificial intelligence is to make a computer simulate a human being in dealing with certainty and uncertainty in information. Logic gives a technique for laying the foundations of this task. Information processing dealing with certain information is based on the classical logic. Nonclassical logic including many-valued logic and fuzzy logic takes the advantage of the classical logic to handle information with various facets of uncertainty, such as fuzziness and randomness. Therefore, nonclassical logic has become a formal and useful tool for computer science to deal with fuzzy information and uncertain information.

For investigation of several properties, such logics have been represented as algebras, that is, sets with one, two, or more algebraic operations satisfying some conditions inspired by these logics. This inspiration is illustrated by the similarities between the names. We have BCK-algebras and a BCK positive logic, BCI-algebras and a BCI positive logic, and BL-algebras and a basic logic.

In many cases, the connection between such algebras and their corresponding logics is much stronger. In this case, one can give a translation procedure which translates all well-formed formulas and all theorems of a given logic L into terms and theorems of the corresponding algebras. In some cases, one can give also an inverse translation. In this case, we say that the given logic and class of such algebras are isomorphic. Nevertheless, the study of algebras motivated by logics is interesting and very useful also in the case when these structures are not isomorphic.

An important class of algebras inspired by logic is BL-algebras introduced by Hajek in order to provide an algebraic proof of the completeness theorem of Basic Logic. A classical example of a BL-algebra is the interval [0,1] endowed with the structure induced by a continuous t-norm. Other important examples of BL-algebras are MV-algebras introduced earlier as a model of some infinitely valued Łukasiewicz logic. All these algebras are also strongly connected with residuated lattices. Hence, the filter theory plays an important role in the study of these algebras. From logic point of view, various filters correspond to various sets of provable formulae in the respective logic.

Below we present several papers on filters in various algebras. In one of these papers, it is proved that there is a one-to-one correspondence between the set of all vague filters and all vague congruence of a lattice implication algebra. In another, the relations among fuzzy t-filters on residuated lattices are described. Another paper is devoted to the filter topology on lattice implication algebras. In further articles is characterized the relationship between the main types of fuzzy filters in BE-algebras and EQ-algebras. The role of ideals and soft sets in BL-algebras is described in another two papers.

In papers on pseudo-weak-R0 algebras, the most simplified axioms system is presented and it is proved that pseudo-weak-R0 algebras are categorically isomorphic to pseudo-IMTL algebras. Pseudo-R0 algebras are categorically isomorphic to pseudo-NM algebras.

Several presented papers are devoted to the possible applications of algebras inspired by logic and fuzzy sets. For example, a robust fuzzy logic controller is proposed for stabilization and disturbance rejection in nonlinear control systems of a particular type. The dynamic feedback controller is designed as a combination of a control law that compensates for nonlinear terms in a control system and a dynamic fuzzy logic controller that addresses unknown model uncertainties and an unmeasured disturbance. A mathematical derivation is used to prove that the controller is able to achieve asymptotic stability by processing state measurements. Properties of some hesitant triangular fuzzy aggregation operators based on Bonferroni means also are discussed. In one paper, the method of a construction of a ranking of the physical matches is proposed. This method is based on the fuzzy clustering analysis.

Decisions are often made by many decision experts having preference of risks. In view of the grey situation group decision-making problems, a grey situation group decision-making method on the basis of prospect theory is shown. The method takes the positive and negative ideal situation distance as reference points, defines positive and negative prospect value function, and introduces decision experts' risk preference into grey situation decision-making to make the final decision more in line with decision experts' psychological behavior. One possibility of determination of the weight of each decision expert sets up comprehensive prospect value matrix for decision experts' evaluation, and finally determination of the optimal situation is presented.

*Jianming Zhan* *Jianming Zhan* *Bijan Davvaz* *Bijan Davvaz* *Wieslaw A. Dudek* *Wieslaw A. Dudek* *Young Bae Jun* *Young Bae Jun* *Hee Sik Kim* *Hee Sik Kim*

